# Examining the Role of Cone-expressed RPE65 in Mouse Cone Function

**DOI:** 10.1038/s41598-018-32667-w

**Published:** 2018-09-21

**Authors:** Alexander V. Kolesnikov, Peter H. Tang, Vladimir J. Kefalov

**Affiliations:** 1 Department of Ophthalmology and Visual Sciences, Washington University School of Medicine, Saint Louis, MO 63110 USA; 2 Department of Ophthalmology, Medical University of South Carolina, Charleston, SC 29425 USA; 3 Present Address: Byers Eye Institute, Department of Ophthalmology, Stanford University School of Medicine, Palo Alto, CA 94303 USA

## Abstract

Efficient chromophore supply is paramount for the continuous function of vertebrate cone photoreceptors. It is well established that isomerization of all-*trans*- to 11-*cis*- retinoid in the retinal pigmented epithelium by RPE65 is a key reaction in this process. Mutations in RPE65 result in a disrupted chromophore supply, retinal degeneration, and blindness. Interestingly, RPE65 has recently been found to also be expressed in cone photoreceptors in several species, including mouse and human. However, the functional role of cone-expressed RPE65 has remained unknown. Here, we used loss and gain of function approaches to investigate this issue. First, we compared the function of cones from control and RPE65-deficient mice. Although we found that deletion of RPE65 partially suppressed cone dark adaptation, the interpretation of this result was complicated by the abnormal cone structure and function caused by the chromophore deficiency in the absence of RPE65 in the pigmented epithelium. As an alternative approach, we generated transgenic mice to express human RPE65 in the cones of mice where RPE65 expression is normally restricted to the pigmented epithelium. Comparison of control (RPE65-deficient) and transgenic (RPE65-expressing) cones revealed no morphological or functional changes, with only a slight delay in dark adaptation, possibly caused by the buffering of retinoids by RPE65. Together, our results do not provide any evidence for a functional role of RPE65 in mouse cones. Future studies will have to determine whether cone-expressed RPE65 plays a role in maintaining the long-term homeostasis of retinoids in cones and their function and survival, particularly in humans.

## Introduction

Our nighttime and daytime visual functions are mediated by two major classes of retinal photoreceptor cells, the rods and cones, respectively. Visual perception begins with the absorption of light by visual pigments within rods (rhodopsin) and cones (cone opsin), which triggers the photoisomerization of their covalently-bound chromophore (11-*cis*-retinal) to its all-*trans* conformation^1^. Activated pigments initiate a cascade of phototransduction reactions that ultimately generates a physiological response to light^2,3^. Bleached photoreceptors restore their photon-catching function by continuous recycling of all-*trans*-retinal back to 11-*cis*-retinal via a process called the visual (retinoid) cycle. In vertebrates, two major interrelated visual cycles have been described. The classical retinal pigmented epithelium (RPE)-based visual cycle supplies 11-*cis*-retinal to both rods and cones, while a more recently discovered alternative visual cycle functions through Müller glial cells of the retina to deliver chromophore specifically to cones^4–7^. An additional mechanism involving direct all-*trans*-retinal photoisomerization to 11-*cis*-retinal with blue light, similar to that occurring within invertebrate opsins, has also been described^8,9^.

A critical step in the classical visual cycle is the enzymatic isomerization of all-*trans*-retinol, released from the photoreceptors, to 11-*cis*-retinal. This reaction takes place in the RPE and is catalyzed by an abundantly expressed 65-kDa protein named RPE65^10–12^. In addition to its crucial role in rod pigment regeneration, RPE65 is also important for the function and survival of human cone photoreceptors^13^. Numerous naturally occurring mutations in the *Rpe65* gene result in a disrupted visual cycle and lead to a severe clinical disease called Type 2 Leber congenital amaurosis. This blinding condition is characterized by the early loss of cone-mediated vision, sensory nystagmus, and the lack of electroretinographic (ERG) signals^14^.

Surprisingly, in addition to its well-known localization within the pigmented epithelium, RPE65 protein has been also found in the cones of numerous mammalian species, including rod-dominant mouse, cow, and human^15–17^ and the cone-dominant lizard^5^. With the exception of lizard cones, where this protein is localized in their inner segments, in all other reported cases RPE65 is present in the cone outer segments (OS), with particular abundance in their basal region^5^. Interestingly, the level of RPE65 within cones across different strains of mice appears to be inversely related to its level in the RPE^16^, suggesting a possible role in the recycling of chromophore in cones.

Unlike its well-established role in the RPE, the function of cone RPE65 remains unclear. As the enzymes required for generating its all-*trans*-retinyl ester substrates (LRAT and ARAT) have not been identified in photoreceptors, cone RPE65 is not likely to operate as a retinoid isomerase. Instead, recent studies have hypothesized its function as a retinoid binding protein which could stabilize 11-*cis*-retinol delivered from Müller cells and shuttle it along the cone OS^16^. In addition, based on *in vitro* experiments with a cone-derived 661 W cell line, another possible role of cone RPE65 could be to bind 11-*cis*-retinyl esters to facilitate their hydrolysis^17^, or even to enhance the oxidation of 11-*cis-*retinol by promoting its interaction with a yet unidentified cone-specific retinol dehydrogenase^18–20^. Interestingly, in contrast to mouse strains with higher levels of cone RPE65 where cone pigment is fully regenerated after dark adaptation, those with little or no cone RPE65 appear to have a mild chromophore deficiency^16^. However, direct biochemical or physiological evidence for a functional role of RPE65 in cones *in vivo* has remained missing.

Here, we used loss and gain of function approaches to investigate the proposed physiological function of cone RPE65 in the alternative visual cycle, as well as its possible role as a structural and functional component of the cone OS that might support the viability and signaling of these photoreceptors under conditions of severe visual chromophore deficiency.

## Results

### Systemic administration of 9-*cis*-retinal partially rescues cone function and morphology in RPE65-deficient mice

In attempt to determine the potential role of RPE65 protein expressed within mouse cones, we first used the available *Rpe65*^−/−^ mice^10^. Expression of both M- and S-opsins, cone density, and function in this model are all severely compromised as early as two weeks after birth^21^, complicating its physiological characterization. However, they can be rescued to a significant degree by systemic injections of exogenous 11-*cis*-retinal or 9-*cis*-retinal, starting before the onset of cone degeneration^21,22^. We applied a previously described sustained delivery method of commercially available 9-*cis*-retinal to mouse photoreceptors *in vivo*, in which a 1:10 (by volume) mixture of the retinoid and a thermosensitive polymer Matrigel^R^ Matrix are repeatedly injected subcutaneously. Matrigel is a growth factors-containing hydrogel which is a liquid at 4 °C and can be loaded with retinoid. Upon injection, Matrigel undergoes a phase change into its solid form at the mouse body temperature, and then it slowly releases the bound chromophore into the circulation, from where it is eventually transported to the eye and retina^23^.

**Figure 1:**
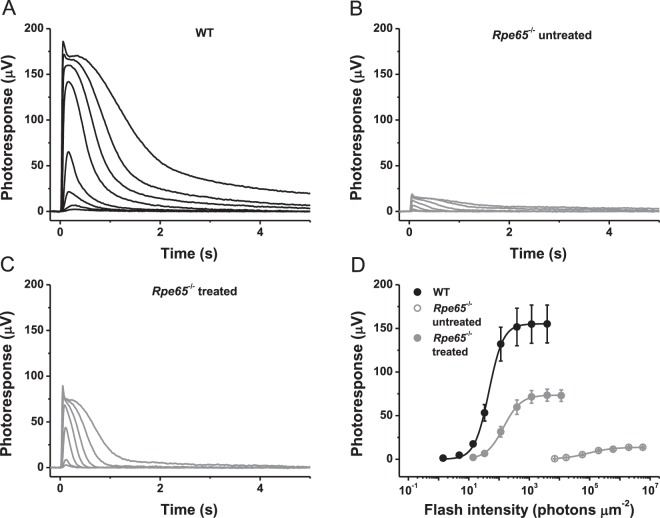
Treatment with 9-*cis*-retinal largely restores sensitivity of chromophore-deficient mouse rods. (**A**) Representative family of transretinal rod ERG responses from wild-type (C57BL/6J) mouse retinas. Test flashes of 505 nm light with intensities of 1.6, 4.8, 14, 33, 114, 392, 1.2 × 10^3^, and 3.9 × 10^3^ photons μm^−2^ were delivered at time 0. (**B**) Representative family of transretinal rod ERG responses from *Rpe65*^−/−^ mouse retinas. Test flashes of 505 nm light with intensities of 6.9 × 10^3^, 1.7 × 10^4^, 5.7 × 10^4^, 2.0 × 10^5^, 6.0 × 10^5^, 2.0 × 10^6^, and 5.7 × 10^6^ photons μm^−2^ were delivered at time 0. There were no responses if lower flash intensities were applied. (**C**) Representative family of transretinal rod ERG responses from retinas of *Rpe65*^−/−^ animals repeatedly injected with 9-*cis*-retinal and Matrigel^R^ Matrix. Test flashes of 505 nm light with intensities of 14, 33, 114, 392, 1.2 × 10^3^, 3.9 × 10^3^, and 1.1 × 10^4^ photons μm^−2^ were delivered at time 0. (**D**) Averaged rod intensity-response functions (mean ± SEM) for wild-type (n = 3), *Rpe65*^−/−^ (n = 4), and 9-*cis*-retinal treated *Rpe65*^−/−^ (n = 7) mice. Error bars for some points are smaller than the symbol size. Hyperbolic Naka-Rushton fits yielded half-saturating intensities (*I*_1/2_) of 46, 8.9 × 10^4^, and 142 photons μm^−2^, respectively.

The efficacy of chromophore administration was first examined in the rods of *Rpe65*^−/−^ mice that do not degenerate appreciably until 7 weeks of age^10^. Retinoid injections were initially performed at postnatal day 10 (P10), just before mice open their eyes, and then at P14, P18, and P23. The animals were raised in constant darkness from the first injection onwards to prevent bleaching of visual pigments and to improve the effectiveness of chromophore supplementation^24^. A cohort of control mice from the same litter was kept dark-reared under similar experimental conditions but without chromophore supplementation. Transretinal (*ex vivo*) rod ERG recordings were carried out at P28–P30 (Fig. 1). In this method, the presence of postsynaptic inhibitors blocked contributions of higher order response components (such as ON bipolar cell-driven ERG *b*-wave), allowing us to isolate the rod photoresponse component^25^. Consistent with previously reported results^26^, chromophore-deficient rods in *Rpe65*^−/−^ mice exhibited ~10-fold smaller and significantly faster responses to test flashes of green light and were desensitized by approximately ~2000-fold, as compared with those from wild-type (C56BL/6J) animals (Fig. 1A,B,D). Systemic administration of Matrigel preloaded with 9-*cis*-retinal largely restored the sensitivity and kinetics of rod responses in this mouse model (Fig. 1A,C,D) thus confirming the efficiency of this delivery method. The remaining ~3-fold difference in rod photosensitivity between chromophore-treated *Rpe65*^−/−^ mice and wild-type animals can be entirely attributed to the correspondingly lower quantum efficacy of vertebrate isorhodopsin (a pigment formed with 9-*cis*-retinal) compared to rhodopsin, in which opsin is bound to 11-*cis*-retinal^27^.

**Figure 2:**
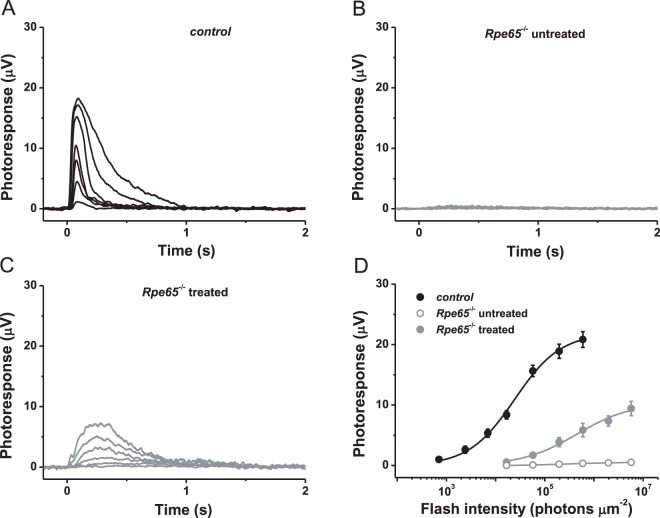
Treatment with 9-*cis*-retinal partially restores sensitivity of chromophore-deficient mouse cones. (**A**) Representative family of transretinal cone ERG responses from control *Rpe65*^+/+^*Gnat1*^−/−^ mouse retinas. Test flashes of 505 nm light with intensities of 705, 2.4 × 10^3^, 7.0 × 10^3^, 1.7 × 10^4^, 5.7 × 10^4^, 2.0 × 10^5^, and 6.0 × 10^5^ photons μm^−2^ were delivered at time 0. (**B**) Representative family of transretinal cone ERG responses from *Rpe65*^−/−^*Gnat1*^−/−^ mouse retinas. Test flashes of 505 nm light with intensities of 1.7 × 10^4^, 5.7 × 10^4^, 2.0 × 10^5^, 6.0 × 10^5^, 2.0 × 10^6^, and 5.7 × 10^6^ photons μm^−2^ were delivered at time 0. (**C**) Representative family of transretinal cone ERG responses from retinas of *Rpe65*^−/−^*Gnat1*^−/−^ animals repeatedly injected with Matrigel loaded with 9-*cis*-retinal. Intensities of test flashes of 505 nm light were the same as in (**B**). (**D**) Averaged cone intensity-response functions (mean ± SEM) for control *Rpe65*^+/+^*Gnat1*^−/−^ (n = 9), *Rpe65*^−/−^*Gnat1*^−/−^ (n = 3), and 9-*cis*-retinal treated *Rpe65*^−/−^*Gnat1*^−/−^ (n = 10) mice. Error bars for some points are smaller than the symbol size. Hyperbolic Naka-Rushton fits yielded half-saturating intensities (*I*_1/2_) of 2.4 × 10^4^ and 4.5 × 10^5^ photons μm^−2^ for control and *Rpe65*^−/−^*Gnat1*^−/−^ animals treated with 9-*cis*-retinal, respectively. Because of their small values, data for untreated *Rpe65*^−/−^*Gnat1*^−/−^ mice were not fitted with Naka-Rushton functions and connected with straight lines instead.

We then applied the same procedure to supply the rapidly degenerating cones in *Rpe65*^−/−^ mice with exogenous 9-*cis*-retinal (Fig. 2). To facilitate cone recordings, these animals were derived on a *Gnat1*^−/−^ background (lacking rod transducin α-subunit) that eliminates the rod component of the light response without affecting cone morphology or function^28^. In this study, we performed all of our physiological experiments using mid-wavelength light signal cone (M-cone) response, due to methodological limitations of using UV light and the proximity of the absorbance spectra of M-cone pigment to that of rhodopsin. This allows for a better comparison between rod and M-cone response. In contrast to rods, M-cones in untreated 4-week-old *Rpe65*^−/−^*Gnat1*^−/−^ mice were essentially unresponsive to green light, indicating the rapid deterioration of their function as compared to cells from control *Gnat1*^−/−^ retinas (Fig. 2A,B,D). Systemic administration of 9-*cis*-retinal/Matrigel mixture only partially restored cone function (Fig. 2C). Although the maximal amplitude of M-cone response recovered to ~50% of that in *Gnat1*^−/−^ mice, the sensitivity of chromophore-treated cones was still ~20 times lower (indicating a 7-fold reduction, considering the use of 9-*cis*-retinal), and the responses revealed abnormal and substantially slower activation and inactivation (Fig. 2A,C,D). The time-to-peak of dim flash M-cone responses in Matrigel-treated animals lacking RPE65 ranged from 250 to 300 ms, as compared to only ~70 ms in control mice.

**Figure 3:**
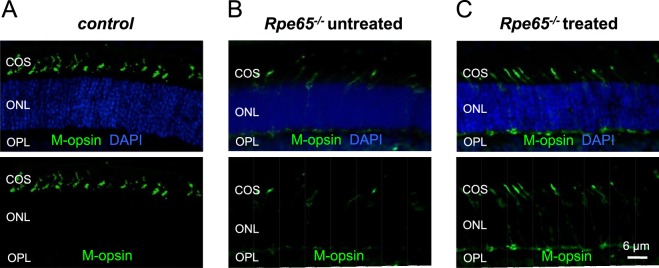
Chromophore treatment corrects the localization of M-cone opsin and the structure of cone outer segments in RPE65-deficient mice. M-opsin immunolocalization (green) in the dorsal retina of control *Rpe65*^+/+^*Gnat1*^−/−^ (**A**), uninjected *Rpe65*^−/−^*Gnat1*^−/−^ animals (**B**), and *Rpe65*^−/−^*Gnat1*^−/−^ mice injected with Matrigel loaded with 9-*cis*-retinal (**C**). COS: cone outer segments; ONL: outer nuclear layer; OPL: outer plexiform layer. Cell nuclei were stained with DAPI (blue). Scale bar, 6 μm.

Opsin mislocalization plays a key role in the pathophysiology of cone loss in diseases involving a disrupted retinoid cycle. Previous studies in RPE65- and LRAT-deficient mice indicate that trafficking of several phototransduction proteins (including M- and S-opsins and G-protein transducin) to the cone OS is impaired in these models, and both opsins become distributed throughout the entire length of the cell^29,30^. Therefore, we characterized the localization of M-opsin in cones of 4-week-old control and 9-*cis*-retinal-treated *Rpe65*^−/−^ animals (Fig. 3). In accordance with previous findings^22^, prolonged supply of visual chromophore to RPE65-deficient mice largely improved targeting of M-opsin to the cone OS in the dorsal retina as compared to untreated counterparts (Fig. 3B,C). Cone numbers and density were also substantially increased in chromophore-treated *Rpe65*^−/−^ animals. However, the rescue was only partial, as some M-pigment was still present in cone synaptic terminals (Fig. 3C). This was in contrast to that observed in *Gnat1*^−/−^ controls, which also had a greater number of dorsal cones overall (Fig. 3A). Together with the delayed cone response kinetics evident from our *ex vivo* ERG recordings described above, this result indicates that even prolonged treatment with exogenous 9-*cis*-retinal cannot rescue fully the trafficking of phototransduction proteins to cone OS and the physiological function of M-cones in RPE65-deficient mice. Despite that, the method still allowed us to investigate how the removal of RPE65 from cones affects their dark adaptation.

### Suppressed dark adaptation of M-cones in mice lacking RPE65

**Figure 4:**
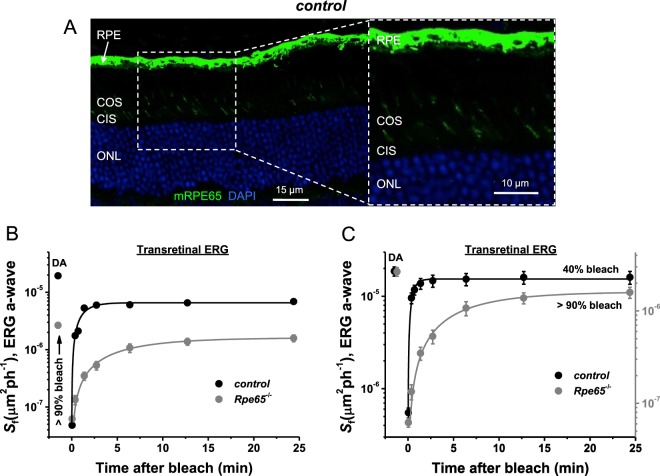
Delayed M-cone dark adaptation in retinas of RPE65-deficient mice. (**A**) Endogenous expression of RPE65 protein in cones of control *Rpe65*^+/+^*Gnat1*^−/−^ mice was confirmed by immunohistochemistry using PETLET RPE65 antibody (green). Cell nuclei were stained with DAPI (blue). RPE: retinal pigmented epithelium; COS: cone outer segments; CIS: cone inner segments; ONL: outer nuclear layer. Scale bar, 15 μm. The inset shows higher magnification of the selected area. Scale bar, 10 μm. (**B**) Recovery of cone ERG *a*-wave flash sensitivity (*S*_f_; mean ± SEM) in isolated retinas of control (n = 9) and *Rpe65*^−/−^*Gnat1*^−/−^ mice injected with Matrigel loaded with 9-*cis*-retinal (n = 12) after bleaching >90% of cone pigment at time 0 with 505 nm LED light. Error bars for some points are smaller than the symbol size. Data were fitted with single-exponential functions that yielded time constants of ~1.2 min and 6.1 min, respectively. *S*_f_^DA^ denotes the sensitivity of dark-adapted cones. (**C**) Comparison of the recovery of cone ERG *a*-wave flash sensitivity (*S*_f_; mean ± SEM) in isolated retinas of control *Rpe65*^+/+^*Gnat1*^−/−^ mice (n = 9, left y-axis) and *Rpe65*^−/−^*Gnat1*^−/−^ littermates injected with Matrigel loaded with 9-*cis*-retinal (n = 12, right y-axis) under conditions of bleaching causing equal initial cone desensitization (1.5 log units). Bleaching ~40% (control *Rpe65*^+/+^*Gnat1*^−/−^) or >90% (*Rpe65*^−/−^*Gnat1*^−/−^) of cone pigment at time 0 was performed with 505 nm LED. Data from *Rpe65*^−/−^*Gnat1*^−/−^ mice were replotted from (**B**). Data were fitted with single-exponential functions that yielded time constants of ~0.5 min and 6.1 min, respectively. The y-axis for the data from mutant mice (right) was arbitrary scaled up to match the initial dark-adapted cone sensitivity (*S*_f_^DA^) of the two strains.

RPE65 has been found in cones of both rod-dominant human and cone-dominant lizard retinas, where it has been proposed to play a role as a facilitator of the cone-specific visual cycle^5,17^. However, this issue has not been examined directly with experimental tools. To address the possible role of cone-expressed RPE65 in mouse cone dark adaptation, we monitored the recovery of M-cone ERG *a*-wave flash sensitivity (*S*_f_) after almost complete (>90%) bleaching of cone visual pigment with 505 nm LED light. Cone responses were obtained by transretinal ERG recordings from isolated mouse retinas, which allowed the pharmacological isolation of the photoreceptor component of the response (Fig. 4). Under these conditions, the classical RPE visual cycle is not functional due to the removal of the RPE, and the recovery of cone sensitivity relies exclusively on chromophore processing within the retina^31^. For this experiment, control *Gnat1*^−/−^ and *Rpe65*^−/−^*Gnat1*^−/−^ mice (derived from breeding of the two individual knockout strains) were used. It should be noted that the original *Rpe65*^−/−^ line^10^ was generated on the C57BL/6J genetic background that has been shown to express RPE65 protein in cones^16^. In contrast, the original *Gnat1*^−/−^ strain^28^ was derived and maintained on the BALB/c background which lacks cone RPE65 expression^16^. Thus, it was important to analyze whether our composite control *Gnat1*^−/−^ strain (*Rpe65*^+/+^*Gnat1*^−/−^) with a mixed C57BL/6J x BALB/c background preserved RPE65 in retinal cones. Indeed, the endogenous expression of mouse cone RPE65 protein in this line was confirmed by immunohistochemistry using PETLET anti-RPE65 antibody (Fig. 4A and its inset, green). This allowed us to compare the dark adaptation of RPE65-expressing cones in control (*Gnat1*^−/−^) retinas and RPE65-deficient cones in *Rpe65*^−/−^*Gnat1*^−/−^ retinas.

We began with control recordings, first determining the dark-adapted sensitivity (*S*_f_^DA^) of 4-week-old control M-cones. The isolated retina was then exposed to bright light that rapidly bleached an estimated >90% of the cone visual pigment. Following this nearly complete pigment bleach, cones in control retinas were initially desensitized by almost 3 log units and then gradually recovered most of their sensitivity in the dark, with a time constant of ~1.2 ± 0.2 min (Fig. 4B). The recovery of cone sensitivity was then measured in retinas of age-matched RPE65-deficient mice (*Rpe65*^−/−^*Gnat1*^−/−^) that were raised in constant darkness and repeatedly supplemented with 9-*cis*-retinal/Matrigel mixture, as described in the previous section. Consistent with those results (Fig. 2D), the initial dark-adapted sensitivity of M-cones in this line was ~10 times lower. We found that the lack of RPE65 decelerated the subsequent cone dark adaptation by ~5-fold (time constant of ~6.1 ± 0.4 min) under identical (>90%) pigment bleaching conditions. Interestingly, the fractional level of cone sensitivity recovery (*S*_f_/*S*_f_^DA^) by 30 min postbleach was ~1.7-fold higher in mutant retinas, as compared to controls (~60% vs. ~35%, respectively). One possible explanation of this effect could be the accumulation of an excess of 9-*cis*-retinal in cones or retinas of chromophore-treated mutant mice, which would itself readily recombine with free opsin after the bleach and increase the relative level of regenerated pigment. However, control experiments with *Gnat1*^−/−^ mice supplemented with the retinoid using the same protocol ruled out this possibility (data not shown). Thus, a second, more likely explanation of the robust final recovery of sensitivity in RPE65-deficient cones is the lower amount of M-opsin in their cone OS, which would require less chromophore for pigment regeneration than in control cones. A lower pigment content in these cones is also consistent with the smaller initial cone desensitization produced by the bleach in RPE65-deficient retinas (1.5 log units) compared to controls (3 log units).

The ~10-fold difference in sensitivity of dark-adapted cones between control *Gnat1*^−/−^ and *Rpe65*^−/−^*Gnat1*^−/−^ mice and the resulting different level of initial bleach-induced desensitization motivated us to perform an additional experiment with control retinas. In this case, we bleached only ~40% of their M-cone pigment to achieve approximately the same extent of initial cone desensitization (~1.5 log units) as that caused by bleaching >90% pigment in mutant retinas (Fig. 4C). Compared in this way, the difference in rates of cone dark adaptation between control and 9-*cis*-retinal treated *Rpe65*^−/−^*Gnat1*^−/−^ mice was even more dramatic (time constants of ~0.5 ± 0.06 min and ~6.1 ± 0.4 min, correspondingly). For better visualization, the data from mutant animals were replotted from Fig. 4B using the right y-axis of Fig. 4C.

Together, these results may indicate that chromophore recycling through the retina visual cycle was compromised when cones were lacking their RPE65 protein. However, the abnormally slow kinetics of cone responses in RPE65-deficient mice (Fig. 2C) and incomplete rescue of cone morphology and function upon treatment with exogenous 9-*cis*-retinal (Figs 2 and 3) could have also contributed to the suppressed dark adaptation of mutant cones, thus complicating the interpretation of the results obtained with this animal model.

### Transgenic expression of RPE65 in normal mouse M-cones

We developed an alternative approach to more directly address the possible role of cone RPE65 in dark adaptation of mammalian cone photoreceptors. To do that, we generated a novel mouse line which transgenically expresses human RPE65 protein in normal retinal cones under the control of the rhodopsin kinase promoter (see Materials and Methods). The choice of a highly homologous human RPE65 (Leu-450 isoform) was mainly dictated by the possibility to distinguish it from the endogenous mouse protein by immunohistochemistry. Again, to facilitate cone recordings, we derived this line on *Gnat1*^−/−^ background. Because proven C57BL/6J mouse embryos were used for ES cells microinjection to generate a single chimeric animal, we further crossed it with original *Gnat1*^−/−^ strain for five generations. This was necessary and sufficient to eliminate any residual cone expression of mouse RPE65 in C57BL/6J mice^16^.

**Figure 5:**
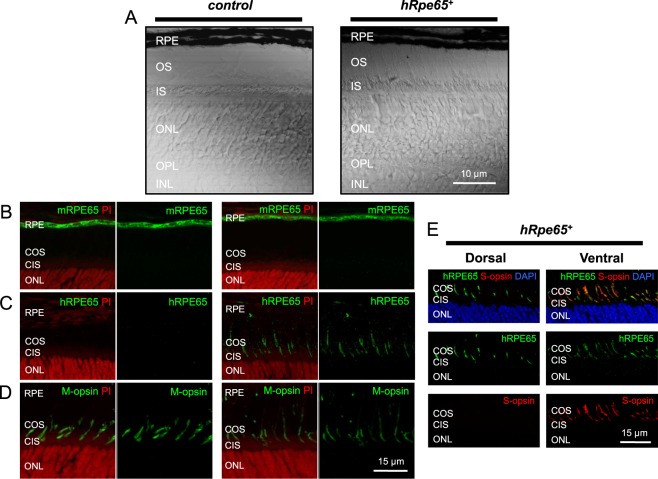
Transgenic expression of human RPE65 in normal cones lacking endogenous RPE65 protein. (**A**) Retinal morphology in control *Gnat1*^−/−^ and transgenic *Gnat1*^−/−^*hRpe65*^+^ mice. Confocal images in transmitted light. RPE: retinal pigmented epithelium; OS: photoreceptor outer segments; IS: photoreceptor inner segments; ONL: outer nuclear layer; OPL: outer plexiform layer; INL: inner nuclear layer. Scale bar, 10 μm. (**B**) Absence of endogenous RPE65 in cones of control *Gnat1*^−/−^ (left) and transgenic *Gnat1*^−/−^*hRpe65*^+^ (right) mice. Expression of mouse RPE65 (mRPE65), examined by immunohistochemistry with PETLET RPE65 antibody (green), was restricted to the RPE. Cell nuclei were stained with propidium iodate (red). RPE: retinal pigmented epithelium; COS: cone outer segments; CIS: cone inner segments; ONL: outer nuclear layer. (**C**) Human RPE65 (hRPE65, DALEED antibody, green) is expressed in retinas of transgenic (right) mice but not in control (left) retinas. Cell nuclei were stained with propidium iodate (red). RPE: retinal pigmented epithelium; COS: cone outer segments; CIS: cone inner segments; ONL: outer nuclear layer. (**D**) Normal cone density and morphology in retinas of control (left) and transgenic (right) animals. Expression of M-opsin was examined by immunohistochemistry with rabbit M-opsin antibody (green). Cell nuclei were stained with propidium iodate (red). RPE: retinal pigmented epithelium; COS: cone outer segments; CIS: cone inner segments; ONL: outer nuclear layer. Scale bar in (**B**–**D**), 15 μm. (**E**) Transgenic human RPE65 (green) is also expressed in ventrally-located retinal S-cones (bottom) that are not present in the far dorsal area of the retina (top), as evidenced by immunostaining with S-opsin antibody (red). Cell nuclei were stained with DAPI (blue). COS: cone outer segments; CIS: cone inner segments; ONL: outer nuclear layer. Scale bar, 15 μm.

As expected, both retina and RPE in transgenic mouse had normal morphological appearance (Fig. 5A). We used two separate RPE65 antibodies to investigate its expression in our transgenic mice: PETLET anti-RPE65 antibody, which recognizes the mouse form of the protein, and DALEED anti-RPE65 antibody, which recognizes specifically the human form but not the mouse form of the protein. Immunostaining of retinal cross-sections from 6-week-old control *Gnat1*^−/−^ animals and transgenic *Gnat1*^−/−^*hRpe65*^+^ littermates with PETLET anti-RPE65 antibody did not reveal the presence of endogenous mouse RPE65 in their cones; however, a normal abundance of RPE65 was shown in the RPE layer (Fig. 5B, green signal, compare this result to the one shown in Fig. 4A). In stark contrast, human RPE65 protein was exclusively expressed in the cone OS of transgenic animals, but not in transgene-negative controls, as was detected by immunostaining with the DALEED anti-RPE65 antibody (Fig. 5C). Surprisingly, the expected abundant expression of human RPE65 in rods (as the *Grk1* promoter has to be active in all retinal photoreceptors) was not observed in our transgenic model. Although the reason for that is currently unclear (for instance, it could be caused by the instability and/or degradation of RPE65 in rods), this circumstance greatly facilitated the detection of transgenic RPE65 protein in mouse cones. Both M-cone density and morphology in the retinas of control and transgenic animals were normal, as was revealed by immunolabeling with M-opsin antibody (Fig. 5D). As expected, transgenic human RPE65 was also expressed in ventrally-located S-cones in *Gnat1*^−/−^*hRpe65*^+^ mice that are not present in the far dorsal part of the retina, as was evidenced by immunostaining with S-opsin antibody (Fig. 5E).

**Figure 6:**
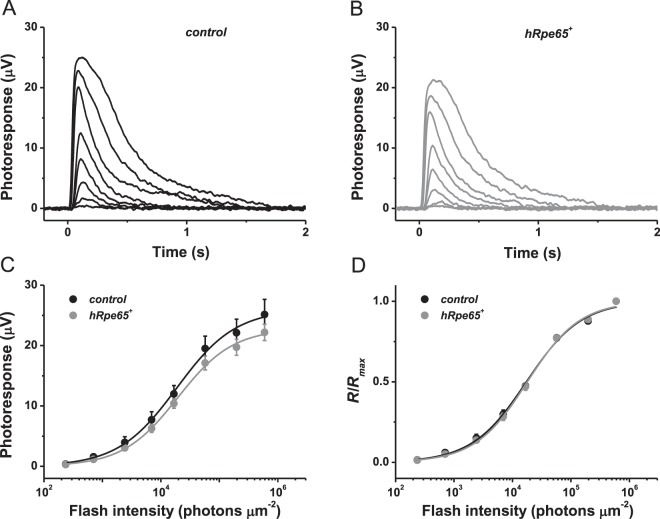
Transgenic expression of human RPE65 does not affect M-cone responses. (**A**) Representative family of *ex vivo* cone ERG responses from control *Gnat1*^−/−^ mouse retinas. Test flashes of 505 nm light with intensities of 235, 705, 2.4 × 10^3^, 7.0 × 10^3^, 1.7 × 10^4^, 5.7 × 10^4^, 2.0 × 10^5^, and 6.0 × 10^5^ photons μm^−2^ were delivered at time 0. (**B**) Representative family of *ex vivo* cone ERG responses from transgenic *Gnat1*^−/−^*hRpe65*^+^ mouse retinas. Test flashes of 505 nm light with the same intensities as in (**A**) were delivered at time 0. (**C**) Averaged cone intensity-response functions (mean ± SEM, not statistically significant at any light intensity) for control *Gnat1*^−/−^ (n = 6) and transgenic *Gnat1*^−/−^*hRpe65*^+^ (n = 6) mice. Hyperbolic Naka-Rushton fits yielded half-saturating intensities (*I*_1/2_) of 1.9 × 10^4^ and 2.0 × 10^4^ photons μm^−2^ for control and transgenic animals, respectively. (**D**) Normalized averaged cone intensity-response relationships (mean ± SEM, no statistical significance at any light intensity) for control *Gnat1*^−/−^ (n = 6) and transgenic *Gnat1*^−/−^*hRpe65*^+^ (n = 6) mice. The data were fitted with Naka-Rushton equations that yielded *I*_1/2_-values as in (**C**). Error bars for some points in (**C**,**D**) are smaller than the symbol size.

To test the possible effect of transgenic expression of human RPE65 on phototransduction cascade in mouse cones, we next performed physiological experiments in perfused retinas from 6-week-old animals. Again, the analysis was limited to M-opsin expressing cones which can be selectively stimulated with visible green light. ERG recordings from isolated retinas in the presence of postsynaptic blockers revealed that under dark-adapted conditions the flash responses of M-cones from *Gnat1*^−/−^*hRpe65*^+^ mice had amplitudes, kinetics and sensitivity comparable to those of responses from control littermates (Fig. 6A–C). The comparable sensitivity of cones with and without RPE65 expression was further evident from fitting normalized averaged cone intensity-response relationships with hyperbolic functions yielding the sensitivity (*I*_1/2_) values of 1.7 × 10^4^ and 1.8 × 10^4^ photons μm^−2^ (*P* > 0.05) for control (with RPE65-negative cones) and transgenic (with RPE65-positive cones) mice, respectively (Fig. 6D). Together with our immunohistochemical analysis, these results demonstrate that the transgenic expression of human RPE65 in mouse M-cones did not produce adverse effects on their viability, overall health, or phototransduction. The lack of detectable morphological or functional changes allowed us to investigate whether cone-expressed RPE65 is important for normal dark adaptation of mammalian M-cones as well as their function in bright light.

### Transgenic expression of RPE65 does not affect pigment regeneration in mouse M-cones

**Figure 7:**
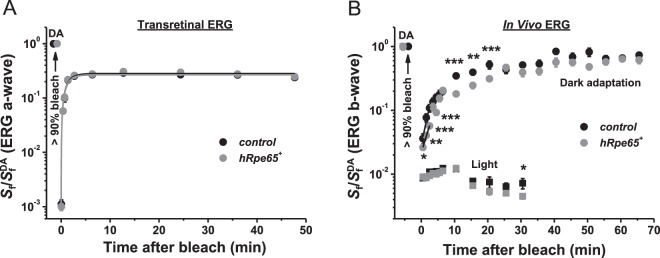
M-cone dark adaptation in transgenic mice expressing human RPE65. (**A**) Recovery of normalized cone *a*-wave flash sensitivity (*S*_f_; mean ± SEM) in isolated retinas of control *Gnat1*^−/−^ (n = 6) and transgenic *Gnat1*^−/−^*hRpe65*^+^ (n = 6) mice after bleaching >90% of cone pigment at time 0 with 505 nm LED light. Data were fitted with single-exponential functions that yielded time constants of ~1.2 min in both cases. *S*_f_^DA^ denotes the sensitivity of dark-adapted cones. (**B**) Recovery of photopic ERG *b*-wave (*S*_f_; mean ± SEM, *P < 0.05, **P < 0.01, ***P < 0.001) *in vivo* in control *Gnat1*^−/−^ (n = 5) and transgenic *Gnat1*^−/−^*hRpe65*^+^ (n = 4) mice after bleaching >90% of cone pigment at time 0 with 520 nm LED light. *S*_f_ was normalized to the corresponding dark-adapted value (*S*_f_^DA^) in each case. Initial rates of *S*_f_ recovery were determined from linear fits (black lines) that yielded the slopes of 0.24 min^−1^ (control) and 0.17 min^−1^ (transgenic). Squares show the change of photopic ERG *b*-wave (*S*_f_; mean ± SEM, *P < 0.05) *in vivo* following illumination with green 530 nm Ganzfeld background light (300 cd m^−2^, 30 min) in control (n = 4) and transgenic (n = 4) mice. *S*_f_ was normalized to the corresponding dark-adapted value (*S*_f_^DA^) in each case. Error bars for some points in (**A**,**B**) are smaller than the symbol size.

To address the possible role of cone-expressed RPE65 in the visual cycle in cones, we first monitored with transretinal ERG recordings the recovery of cone ERG *a*-wave flash sensitivity (*S*_f_) after bleaching >90% of M-cone visual pigment. The experimental procedures were identical to those described above (Fig. 4B). We found that transgenically expressed human RPE65 did neither enhance nor suppress M-cone dark adaptation in isolated mouse retinas which, regardless of its presence, followed a single-exponential timecourse with the time constant of ~1.2 ± 0.2 min (Fig. 7A). The final recovery of cone sensitivity reached ~30% in both cases.

We then performed a similar bleaching experiment in live animals where the cones were in their native environment and the combined action of the retina and RPE visual cycles contributed to their dark adaptation, as demonstrated previously^32^. In this case, the effect of transgenic expression of human RPE65 on the restoration of M-cone photosensitivity after >90% pigment bleach was tested by full-field ERG recordings performed *in vivo*. We used cone ERG *b*-waves to monitor postbleach cone sensitivity (*S*_f_) changes due to very small amplitudes of photopic ERG *a*-waves in mice that are masked by much larger *b*-waves of inverse polarity representing the response of cone bipolar cells. We found that the initial phase of sensitivity recovery, driven by the retina visual cycle^31^ was not accelerated in *Gnat1*^−/−^*hRpe65*^+^ animals (Fig. 7B, circles). Instead, somewhat surprisingly, in the presence of cone RPE65 some data points of the recovery within first the 20 min after the bleach (marked with stars indicating statistical significance) lagged behind the control data. Linear fits to the first three postbleach recovery points (0.5–3 min) yielded rates of 0.24 min^−1^ and 0.17 min^−1^ for RPE65 transgene-positive and negative mice, respectively (Fig. 7B, black lines). Consistent with the idea that the presence of cone RPE65 does not change the turnover of visual chromophore in the RPE, the second, slow component of M-cone dark adaptation (25–65 min) and the final level of cone sensitivity recovery after the bleach were both unaltered in transgenic line. Thus, human RPE65 protein expressed in mouse M-cones lacked the ability to enhance dark adaptation of these photoreceptors, either in isolated retinas or intact eyes.

To test the possibility that transgenic RPE65 could modulate the function of cones under continuous bright light *in vivo*, we performed an additional ERG experiment. After recording dark-adapted M-cone *b*-wave flash sensitivity, green Ganzfeld light (300 cd m^−2^, estimated to bleach ~0.8% M-cone pigment s^−1^) was applied for 30 min. It induced an immediate ~2 log unit light-adaptation of cones which was followed by transient increase in sensitivity (presumably, resulting from the retina network adaptation) of comparable size for control and transgenic animals (Fig. 7B, squares). This initial rise was followed by a short phase of sensitivity stability observed at 5–10 min and then a subsequent gradual sensitivity decline over time. The initial reduction in cone sensitivity was comparable in the two groups of mice. Furthermore, a similar reduction of cone sensitivity was observed after up to 25 min of illumination in both groups of mice. However, after 30 min of light exposure the sensitivity of RPE65 transgenic cones declined significantly compared to that in control cones (*P* < 0.05). Thus, transgenic RPE65 did not improve (and, instead, slightly compromised) the maintenance of M-cone sensitivity under steady bright light conditions.

### Transgenic expression of RPE65 in M-cones of RPE65-deficient mice does not improve their morphology or function

Although our results demonstrate that the transgenic expression of human RPE65 in normal mouse cones does not enhance the recycling of 11-*cis*-retinal through the intraretinal visual cycle, it is still possible that this protein could play an important structural role in species whose cones express it naturally. Therefore, we further investigated if transgenic cone-specific expression of RPE65 would retard or even prevent the well-known degeneration of cones in a chromophore-deficient mouse model. To do that, we generated transgenic animals lacking endogenous RPE65 (*Rpe65*^−/−^*Gnat1*^−/−^*hRpe65*^+^) along with control littermates (*Rpe65*^−/−^*Gnat1*^−/−^) and investigated whether M-cone viability and function were affected by the expression of RPE65 in their cones.

**Figure 8:**
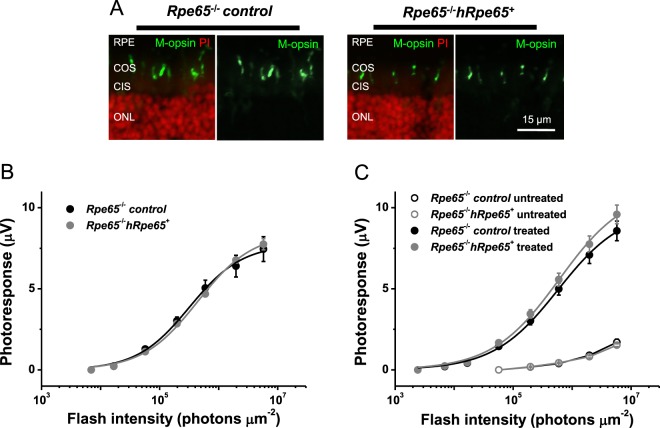
Transgenic expression of human RPE65 in M-cones of RPE65-deficient mice does not improve their morphology or function. (**A**) Comparable cone density and morphology in retinas of control *Rpe65*^−/−^*Gnat1*^−/−^ (left) and transgenic *Rpe65*^−/−^*Gnat1*^−/−^*hRpe65*^+^ (right) mice. M-opsin expression in dorsal retina was examined by immunostaining with rabbit M-opsin antibody (green). Cell nuclei were stained with propidium iodate (red). RPE: retinal pigmented epithelium; COS: cone outer segments; CIS: cone inner segments; ONL: outer nuclear layer. Scale bar, 15 μm. (**B**) Averaged cone intensity-response functions (mean ± SEM, no statistical significance at any point) for control (n = 4) and transgenic (n = 6) retinas treated acutely with exogenous 9-*cis*-retinal. Hyperbolic fits yielded half-saturating intensities (*I*_1/2_) of 3.2 × 10^5^ and 4.4 × 10^5^ photons μm^−2^ for control and transgenic animals, correspondingly. Mice were raised in normal light/dark cycling conditions before retina isolation and chromophore treatment. (**C**) Averaged cone intensity-response functions (mean ± SEM, no statistical significance at any light intensity) for untreated animals (open symbols) or dark-reared mice repeatedly injected with 9-*cis*-retinal and Matrigel (closed symbols). Control (untreated, n = 8; treated, n = 16), transgenic (untreated, n = 8; treated, n = 17). Hyperbolic fits yielded *I*_1/2_-values of 5.8 × 10^5^ and 6.1 × 10^5^ photons μm^−2^ for chromophore-treated control and transgenic animals, respectively. Because of their small values, data for untreated mice (open symbols) were not fitted with Naka-Rushton functions and connected with straight lines instead. Error bars for some points in (**C**) are smaller than the symbol size.

Immunostaining of dorsal retinal cross-sections with M-opsin specific antibody did not show increased density or improved overall cone morphology in 4-week-old RPE65-deficient mice expressing transgenic human RPE65 protein that were raised in normal light/dark cycling conditions, as compared to control animals (Fig. 8A). Consistent with this result, the acute treatment of isolated retinas from transgenic mice with exogenous 9-*cis*-retinal did not reveal improved M-cone response amplitude or photosensitivity over these of controls (Fig. 8B; see Fig. 2D, for comparison). Finally, systemic administration of 9-*cis*-retinal/Matrigel mixture to dark-reared *Rpe65*^−/−^*Gnat1*^−/−^*hRpe65*^+^ animals (using the same method described above) did not improve their cone function beyond the level observed in non-transgenic RPE65-deficient littermates (Fig. 8C, closed symbols). In both cases, the treatment with chromophore partially rescued the cone response when compared to chromophore-untreated control mice, as expected (Fig. 8C, open symbols). Overall, these physiological findings are in a close agreement with the failure of transgenically expressed human RPE65 to prevent or retard the rapid degeneration of M-cones occurring in RPE65-deficient mice.

## Discussion

The role of RPE65 in recycling visual chromophore in the RPE is now firmly established. As the chromophore released from photoreceptors after bleaching their visual pigment is all-*trans*-retinol (Vitamin A), its RPE65-driven reisomerization back to 11-*cis*- retinoid is critical for maintaining continuous photoreceptor function. Similarly, RPE65 is required for the conversion of Vitamin A, taken up from the choroidal vasculature into 11-*cis*-retinal for use in photoreceptors. In the absence of this enzyme, photoreceptors are deprived of chromophore, unable to detect light, and eventually degenerate^16,22,23,29^. In contrast to this well-established and appreciated role of RPE65 within the RPE, its role when expressed in cone photoreceptors has remained unknown. One intriguing possibility is that cone-expressed RPE65 enables recycling of retinoids within cone photoreceptors and contributes to the rapid regeneration of cone visual pigment. However, experiments with amphibian and mouse cones have clearly shown that cones are not able to regenerate their visual pigment when isolated from the retina and RPE^33–35^. Thus, an alternative hypothesis for its function is that it serves as a retinoid binding protein that facilitates the turnover of chromophore in the OS of cones, either by accelerating its release and uptake from RPE and Muller cells, or by stabilizing its 11-*cis*- conformation, thus protecting it from photo- and thermal decay^5,16,17^. Thus, here we performed an extensive set of experiments to investigate the role of cone-expressed RPE65 in the pigment regeneration and dark adaptation of mouse cones.

As cone-expressed RPE65 was expected to affect specifically the turnover of chromophore in cones, we investigated how the absence or presence of RPE65 in cones affects cone dark adaptation. Slowing down chromophore removal or resupply to cones would be expected to delay their dark adaptation. Conversely, accelerating chromophore turnover or protecting the retinoid from isomerization or oxidation would be expected to accelerate or enhance cone dark adaptation. Based on these premises, we used two approaches to address the role of cone-expressed RPE65 in the function of these photoreceptors. First, we compared the function of cones in isolated retinas from control (RPE65-expressing) and *Rpe65*^−/−^ (RPE65-deficient cones) mice. Second, we compared the function of cones where RPE65 was introduced transgenically and cones of mice that normally only express it in RPE cells.

Recordings from isolated retinas revealed that *Rpe65*^−/−^ cones desensitize less and recover 5-fold slower than controls following exposure to bright bleaching light (Fig. 4B). The difference in dark adaptation between control cones and cones from *Rpe65*^−/−^ retinas became even more pronounced when the bleach of the control cells was adjusted to produce comparable levels of initial desensitization (Fig. 4C). One potentially exciting interpretation of these results is that RPE65 in cones serves to accelerate their visual cycle and speeds up cone dark adaptation. However, this analysis was complicated by the chromophore deficiency in *Rpe65*^−/−^ mice caused by the lack of a functional RPE visual cycle. This resulted in cone opsin mislocalization and degeneration, combined with largely suppressed cone function (Figs 2 and 3). While treating the mice with exogenous 9-*cis*-retinal and raising them in darkness ameliorated these problems, cones from *Rpe65*^−/−^ mice still had abnormally slower photoresponses and were about 10-fold less sensitive than controls (Fig. 2). In addition, while improved, cone opsin mislocalization and cone loss were still present in *Rpe65*^−/−^ mice even after the treatment with chromophore (Fig. 3). These issues ultimately prevented us from drawing conclusions about the role of cone-expressed RPE65 based on the loss of function experiments from *Rpe65*^−/−^ animals.

To avoid issues associated with the chromophore deficiency of *Rpe65*^−/−^ mice, we resorted to our second, gain of function, approach and introduced human RPE65 in the cones of mice where it normally is expressed only in the RPE. Our choice of human transgenic RPE65 was dictated by the need to distinguish it from the endogenous mouse protein by immunohistochemistry. The high level of homology (95%) between the human and mouse retinoid isomerases^36^ and the preservation of all key structural elements in human RPE65 as compared to its mouse counterpart allowed us to introduce a very similar protein to mouse cones which was properly localized to their outer segments (Fig. 5C). The transgenic expression of RPE65 in cones did not affect their health or function in dark-adapted conditions (Figs 5 and 6), allowing us to more reliably evaluate its role in cone dark adaptation. Contrary to the results from *Rpe65*^−/−^ mice, we found that introducing RPE65 in cones of a mouse strain that normally lacks cone RPE65 resulted in a slight delay in their dark adaptation *in vivo*, as well as in somewhat larger desensitization of the cones in the presence of steady bright background light (Fig. 7). Both of these results are consistent with the notion that RPE65 in mouse cones causes a minor delay in the regeneration of their visual pigment. Thus, cone RPE65 does not appear to enhance chromophore recycling or have an obvious beneficial effect for the function of these photoreceptors in mice. Consistent with this notion, expressing RPE65 in the cones of *Rpe65*^−/−^ animals also did not improve cone opsin mislocalization or cone function (Fig. 8). The slight delay in cone pigment regeneration in RPE65 transgenic cones might be due to some buffering of retinoids by RPE65.

In summary, our results do not provide any evidence for a beneficial role of RPE65 in mouse cones. However, even though the visual cycle in these cells does not seem to be enhanced in the short term by the presence of RPE65, it is still possible that this protein could play a role in maintaining the long-term homeostasis of retinoids in the cones and enhance in this way the function and survival of these photoreceptors. Furthermore, although we found no effect by RPE65 in mouse cones, this protein could modulate cone function in other species, particularly in humans. A key difference between the retinas of mouse and human is the presence of a cone-rich foveal region in the diurnal human retina, which is absent in the nocturnal mouse. Thus, our findings do not rule out a role for cone-expressed RPE65 in modulating the function of the densely-packed foveal cones and, therefore, supporting the daytime high accuracy vision that these photoreceptors mediate in humans.

## Materials and Methods

### Animals

All experimental protocols were in accordance with the Guide for the Care and Use of Laboratory Animals and were approved by the Washington University Animal Studies Committee. Wild-type mice with a C57BL/6J background were obtained from The Jackson Laboratory (Bar Harbor). Mice with a knockout of the retinal pigmented epithelium protein 65 kDa gene (*Rpe65*^−/−^) were described previously^10^. Rod transducin *α*-subunit knockout (*Gnat1*^−/−^) mice having no rod signaling^28^ were used as controls in all cone physiological experiments. *Rpe65*^−/−^*Gnat1*^−/−^ animals and their littermate *Gnat1*^−/−^ controls were generated by crossing *Rpe65*^−/−^ and *Gnat1*^−/−^ lines. All mice with wild-type allele of *Rpe65* used in this study were homozygous for its Leu450 isoform, as determined using genotyping protocol published elsewhere^37^.

Transgenic mice expressing human RPE65 protein in their cones (*hRpe65*^+^) were generated by the Molecular Genetics Core at Washington University, as follows. The transgene vector was constructed using standard cloning methods^38^. The first step was the removal of *Grk1* (rhodopsin kinase) promoter from *AAV-Grk1-EGFP* plasmid (as an *ApaI–EagI* fragment that included *SV40 SD/SA* sequence), followed by its insertion into *hRpe65-cDNA* plasmid (a gift from Dr. Jian-Xing Ma, University of Oklahoma Health Sciences Center) linearized with *ApaI–NotI*. The final step was the retrieval of the *Grk1-hRpe65* fragment from *Grk1-hRpe65-cDNA* plasmid with *PmeI* and its insertion into the *pI* vector (a modified *pCI* vector containing *SV40 late PolyA* sequence, Promega) cut with *EcoRV–SmaI*. The total size of the final *Grk1(SV40 SD/SA)-hRpe65(SV40 late PolyA)* sequence was ~2.4 kb.

The transgene vector was then electroporated into 129 × 1 Sv/J ES cells (SCC10 line, Siteman Cancer Center Embryonic Stem Cell Core at Washington University) and the recombination event in selected G418-resistent clones was confirmed by Southern blotting and PCR. Positive ES cells were microinjected into C57BL/6J mouse embryos that were then implanted into surrogate C57BL/6J mothers. The resulting chimeras were bred with C57BL/6J mice to generate transgenic *hRpe65*^+^ mice. These animals were further bred with *Gnat1*^−/−^ mice lacking cone expression of endogenous RPE65 for five generations, to obtain *Gnat1*^−/−^*hRpe65*^+^ and control *Gnat1*^−/−^ littermates. Alternatively, *hRPE65*^+^ mice were mated with *Rpe65*^−/−^*Gnat1*^−/−^ animals and further backcrossed to generate *Rpe65*^−/−^*Gnat1*^−/−^*hRpe65*^+^ and control *Rpe65*^−/−^*Gnat1*^−/−^ lines. Genotyping for the presence of *hRpe65* transgene in each generation, as well as that for *Gnat1* and *Rpe65* alleles, was performed according to protocols designed by Transnetyx, Inc.

Young adult animals of either sex (4–6-week-old) were used in all experiments. Animals were provided with standard chow (LabDiet 5053; LabDiet, Purina Mills). All mice with wild-type allele of RPE65 were maintained under a 12 h light (10–20 Lux)/12 h dark cycle and dark-adapted overnight prior to physiological recordings. Unless stated otherwise, all animals with *Rpe65*^−/−^ background were dark-reared from postnatal day 10 (P10) until they reached the age of 4 weeks. For all experiments in this study, animals were euthanized by asphyxiation with rising concentration of CO_2_.

### Antibodies

The PETLET polyclonal rabbit antibody to detect immunoreactive mouse RPE65 protein has been described previously^15^ and was used at a concentration of 2 µg/ml for IHC analysis. The DALEED polyclonal rabbit antibody to detect human RPE65 protein^39^ was used at a dilution of 2 µg/ml for IHC. The anti-M-cone opsin antibody (0.5 µg/ml) was obtained from Millipore. The anti-S-cone opsin antibody (0.4 µg/ml) was from Santa Cruz Biotechnology. Alexa-488 and Alexa-594 secondary antibodies (0.1 µg/ml) were from Invitrogen.

### Immunohistochemistry

After removal of the cornea and lens, the remaining mouse eyecup was fixed in freshly prepared 4% paraformaldehyde in 0.1 M phosphate buffered saline (PBS) at pH 7.4 for 1.3–2 h at room temperature (RT). The eyecup was then washed once in PBS for 10 min and incubated in 30% sucrose buffered with PBS at 4 °C overnight. Next, the eyecup was embedded in Optimal Cutting Temperature compound (Ted Pella), flash-frozen in 2-methylbutane (Sigma) on dry ice, and cut with a cryo-microtome to produce 8 µm sections in dorsal-to-ventral direction through the optic nerve head. Sections were dried for 30 min at RT, gently washed in deionized water for 10 min, dried again for 10 min at RT, and blocked for 1 h at RT with a solution containing 1% bovine serum albumin (Sigma), 1.5% goat serum, and 0.1–0.25% Triton X-100 (Sigma) in PBS. Sections were then incubated at 4 °C overnight with appropriate primary antibodies diluted in a solution containing 1% Tween-20 (Bio-Rad) and 1% Triton X-100 in PBS. Next, sections were washed once in PBS and then incubated with secondary antibodies diluted in PBS containing propidium iodide or DAPI (Invitrogen) for 2 h at RT, washed with PBS (2X, 10 min, RT), and mounted on cover slips with Fluoromount-G (Thermo Scientific) for analysis by confocal microscopy (Olympus FV1000 Confocal Microscope). Images were acquired in both directions from the central region of the retina near the optic nerve head. Immunohistochemistry experiments were typically performed on 2–3 separate animals, and representative images are shown in the respective figures.

### Retinoid delivery

Retinoids were handled under dim red light. A single dose of 9-*cis*-retinal (Sigma) in the amount of 0.5–0.75 µg per animal was dissolved in 20 µl of absolute ethanol and combined with 180 µl of Matrigel^R^ Matrix (Corning) at 4 °C for a final volume of 200 µl^23^. The sample was mixed carefully and injected subcutaneously with a cooled 1 ml syringe into the dorsal torso region of mice with *Rpe65*^−/−^ background at P10. From this point on, animals were placed in constant darkness for dark-rearing and injections were repeated at P14, P18, and P23. Mice were sacrificed and used at P28–P30.

Acute application of exogenous 9-*cis*-retinal to isolated retinas from 4-week-old dark-adapted control and transgenic mice with *Rpe65*^−/−^ background (in this case, animals were raised in normal dark/light cycling conditions until P28) was performed as follows: 300 μg of dried retinoid was dissolved in 8 μl of absolute ethanol and diluted to 8 ml with L15 solution (Sigma) containing 1% lipid-free BSA. The final concentrations of 9-*cis*-retinal and ethanol were ~130 μM and 0.1%, respectively. Before transfer to the perfusion chamber for recordings, a whole retina mounted on filter paper was incubated in a Petri dish with 4 ml of this oxygenated solution for 1 h in the dark at RT.

### Transretinal (*ex vivo* ERG) recordings from isolated retinas

Mice were dark-adapted overnight or dark-reared from P10 (in the case of *Rpe65*^−/−^ mice), and a whole retina was removed from each mouse eyecup under infrared illumination and stored in oxygenated aqueous L15 (13.6 mg/ml, pH 7.4, Sigma) solution containing 0.1% BSA at RT. The retina was mounted on filter paper with the photoreceptor side up and placed into a perfusion chamber between two electrodes connected to a differential amplifier^25,40^. The preparation was perfused with Locke’s solution containing 112.5 mM NaCl, 3.6 mM KCl, 2.4 mM MgCl_2_, 1.2 mM CaCl_2_, 10 mM HEPES, pH 7.4, 20 mM NaHCO_3_, 3 mM Na succinate, 0.5 mM Na glutamate, 0.02 mM EDTA, and 10 mM glucose. This solution was supplemented with 2 mM L-glutamate and 10 µM DL-2-amino-4-phosphonobutyric acid to block postsynaptic components of the photoresponse^41^, and with 20 µM BaCl_2_ to suppress the slow glial PIII component^42^. The perfusion solution was continuously bubbled with a 95% O_2_/5% CO_2_ mixture and heated to 36–37 °C.

Light stimulation was applied by 20 ms test flashes from a calibrated 505 nm LED light. The stimulating light intensity was controlled by a computer in 0.5 log unit steps. Intensity-response relationships were fitted with Naka-Rushton hyperbolic functions, as follows:

$$\begin{array}{ccc}R & = & \frac{{R}_{max}\cdot {I}^{n}}{{I}^{n}\,+\,{I}_{1/2}^{n}},{\rm{for}}\,{\rm{raw}}\,\mathrm{data},\,{\rm{or}}\\ \frac{R}{{R}_{max}} & = & \frac{{I}^{n}}{{I}^{n}\,+\,{I}_{1/2}^{n}},{\rm{for}}\,{\rm{normalized}}\,{\rm{data}},\end{array}$$

where *R* is the transient-peak amplitude of the response, *R*_*max*_ is the maximal response amplitude, *I* is the flash intensity, *n* is the Hill coefficient (exponent), and *I*_1/2_ is the half-saturating light intensity. In experiments designed to monitor the post-bleach recovery of cone *a*-wave flash sensitivity (*S*_f_, see definition below), >90% or ~40% of M-cone visual pigment was bleached with a 3 s exposure to 505 nm light. The bleached fraction was estimated from the following equation:

$$F=1-{e}^{(-I\cdot P\cdot t)},$$

where *F* is the fraction of pigment bleached, *t* is the duration of the light exposure (s), *I* is the bleaching light intensity of 505 nm LED light (1.6 × 10^8^ photons µm^−2^ s^−1^), and *P* is the photosensitivity (7.5 × 10^−9^ µm^2^) of mouse cones at the wavelength of peak absorbance^43^. Photoresponses were amplified by a differential amplifier (DP-311, Warner Instruments), low-pass filtered at 300 Hz (8-pole Bessel), digitized at 1 kHz and stored on a computer for further analysis. Cone *a*-wave flash sensitivity (*S*_f_) was calculated from the linear part of the intensity-response curve, as follows:

$${S}_{f}=\frac{R}{{R}_{max}\cdot I},$$

where *R* is the cone *a*-wave dim flash response amplitude, *R*_*max*_ is the maximal response amplitude for that retina, and *I* is the flash strength. Data were analyzed with Clampfit 10.4 and Origin 8.5 software.

### Electroretinography (ERG)

Dark-adapted control *Gnat1*^−/−^ and transgenic *Gnat1*^−/−^*hRpe65*^+^ mice were anesthetized with an intraperitoneal injection of a mixture of ketamine (100 mg/kg) and xylazine (20 mg/kg). Pupils were dilated with a drop of 1% atropine sulfate. Mouse body temperature was maintained at 37 °C with a heating pad. ERG responses were measured from both eyes by contact corneal electrodes held in place by a drop of Gonak solution. Full-field ERGs were recorded with the UTAS BigShot apparatus (LKC Technologies) using Ganzfeld-derived test flashes of calibrated green 530 nm LED light (within a range from 0.24 cd∙s m^−2^ to 7.45 cd∙s m^−2^). Cone *b*-wave flash sensitivity (*S*_f_, calculated similarly to the *a*-wave *S*_f_ in *ex vivo* ERG recordings) was first determined in the dark (from the average of up to 20 flash responses) and normalized to the maximal *b*-wave amplitude obtained with the brightest white light stimulus of the Xenon Flash tube (700 cd∙s m^−2^). The M-cone pigment was then bleached near completely by a 35 s exposure to bright light delivered by a 520 nm LED focused at the surface of mouse eye cornea that produced ~1.3 × 10^8^ photons µm^−2^ s^−1^. The bleaching fraction, *F*, was estimated by the formula defined above. After the bleach, the recovery of cone *b*-wave *S*_f_ was followed in darkness for up to 1 h. Mice were re-anesthetized with a smaller dose of ketamine (~1/2 of the initial dose) in the middle of that period.

In a subset of experiments, bright green background Ganzfeld illumination (530 nm, 300 cd m^−2^, estimated to bleach ~0.8% M-cone pigment s^−1^) was applied continuously for 30 min to dark-adapted animals, and the cone *b*-wave *S*_f_ change was monitored during that period of light exposure.

### Statistics

For all experiments, data were expressed as means ± SEM. Unless stated otherwise, data were analyzed using the independent two-tailed Student’s *t*-test, with an accepted significance level of *P* < 0.05.
